# Understanding the science-learning environment: A genetically sensitive approach

**DOI:** 10.1016/j.lindif.2012.07.018

**Published:** 2013-02

**Authors:** Claire M.A. Haworth, Oliver S.P. Davis, Ken B. Hanscombe, Yulia Kovas, Philip S. Dale, Robert Plomin

**Affiliations:** a King's College London, Social Genetic and Developmental Psychiatry Centre, United Kingdom; b Department of Psychology, Goldsmiths, University of London, United Kingdom; c Department of Speech and Hearing Sciences, University of New Mexico, United States

**Keywords:** Behavioural genetics, Learning environments, Science ability, Twins, Gene–environment correlation

## Abstract

Previous studies have shown that environmental influences on school science performance increase in importance from primary to secondary school. Here we assess for the first time the relationship between the science-learning environment and science performance using a genetically sensitive approach to investigate the aetiology of this link. 3000 pairs of 14-year-old twins from the UK Twins Early Development Study reported on their experiences of the science-learning environment and were assessed for their performance in science using a web-based test of scientific enquiry. Multivariate twin analyses were used to investigate the genetic and environmental links between environment and outcome. The most surprising result was that the science-learning environment was almost as heritable (43%) as performance on the science test (50%), and showed negligible shared environmental influence (3%). Genetic links explained most (56%) of the association between learning environment and science outcome, indicating gene–environment correlation.

## Highlights

► The science-learning environment is moderately heritable. ► Shared genes explain most of the correlation between environment and performance. ► These results indicate gene–environment correlation.

## 1 Introduction

An understanding of gene–environment interplay will be central in translating behavioural genetic findings into educational implications and in providing evidence for changes to educational policy and practice (Bates, 2008; Grigorenko, 2007; Haworth & Plomin, 2011; Sternberg & Grigorenko, 1999). The strongest evidence for the role of environmental factors comes from genetically sensitive studies that tease apart the effects of nature and nurture and allow the investigation of correlations and interactions *between* nature and nurture (Plomin & Bergeman, 1991; Rutter, Pickles, Murray, & Eaves, 2001). We are not aware of any research that has used this strategy to examine environmental influences on science performance.

Twin analyses allow the investigation of *what* influences the environmental experience and *how* this experience is related to outcomes. The ‘environment’ can be treated as a dependent variable and its variance decomposed into genetic and environmental sources using the differential correlation between identical and fraternal twin pairs. Many ostensible measures of the environment are in fact moderately genetically influenced (Hanscombe, Haworth, Davis, Jaffee, & Plomin, 2010; Kendler & Baker, 2007; Plomin & Bergeman, 1991), indicating gene–environment correlation, that is, a correlation between genetic influences and environmental exposures and experiences (Plomin, DeFries & Loehlin, 1977; Scarr & McCartney, 1983). This arises because individuals influence their environments, for example, by eliciting responses from others or by actively seeking out experiences.

Using the twin design it is also possible to decompose the covariance between an outcome and the environment into genetic and environmental sources. Doing so elucidates the mechanisms by which the environment is related to the outcome. Research in non-genetically sensitive samples typically assumes that environments affect outcomes via environmental pathways. Embedding environmental research within a genetically sensitive study allows us to formally test whether the environment–outcome relationship is in fact environmentally mediated (Rutter et al., 2001).

### 1.1 Environmental influences on school science performance

Previous research has shown that environmental influences are more important for individual differences in science performance than for other academic abilities (Haworth, Kovas, Dale, & Plomin, 2008). The importance of the environment also appears to increase with age (Haworth, Dale, & Plomin, 2009), making science performance in adolescence a pertinent target for the identification of the specific environmental influences that are involved. School science performance is influenced by both shared and non-shared environmental factors; at age 12 shared environmental influences accounted for 32% of the variance in performance, and non-shared environments accounted for a further 21% (Haworth et al., 2009).

### 1.2 School learning environments

The role of the teacher and the classroom environment on educational achievement has been an important topic in educational research (Fraser, 1998; Fraser & Walberg, 1981), and more recently the peer and home learning environments have been a focus of research (Fraser & Kahle, 2007). Reports consistently demonstrate an influence of the classroom on achievement, particularly in analyses that focus on average effects across classrooms. Analyses of individual-level effects have also demonstrated the role of the classroom environment on achievement, albeit with smaller correlations. For example, individual-level associations between classroom environment and science achievement were found to be 0.13 in a sample of more than 3000 students (Fraser & Kahle, 2007), while the peer environment correlated even less with science achievement. The current analyses considered these classroom and peer measures within a genetically sensitive design to understand the genetic and environmental aetiology of learning environments and their links to science performance. Given the interest in sex differences in science that typically emerge in adolescence (Halpern et al., 2007), we were also interested to assess any sex-specific environmental effects.

## 2 Methods

### 2.1 Sample

The Twins Early Development Study (TEDS) is a study of twins born in England and Wales between 1994 and 1996 (Oliver & Plomin, 2007). TEDS is reasonably representative of the general population in terms of parental education, ethnicity and employment status (Kovas, Haworth, Dale, & Plomin, 2007). Zygosity was assessed through a parent questionnaire of physical similarity (Price et al., 2000). For cases where zygosity was unclear, DNA testing was conducted.

TEDS families were invited to participate in the 14-year study, which included a web-based battery of cognitive tests, and postal questionnaires. Parents provided informed consent for each assessment. The mean age at assessment was 14.03 (sd = 0.60) for the web-based tests; and 14.07 (sd = 0.56) for the questionnaire. Not all families provided data for both assessments; see Table 1 for the number of complete twin pairs for each measure.

### 2.2 Measures

#### 2.2.1 The learning environment

To assess the science-learning environment we used items from the Classroom, Home and Peer Environment Influences Scale (Fraser & Kahle, 2007). We included items from the classroom and the peer domains to assess the support given by both teachers and peers in the science-learning environment. The questionnaire included 10 of the original 12 items scored on a five-point scale from ‘almost never’ to ‘very often’. To assess the classroom environment there were 6 items primarily focused on interactions between the students and the teacher while in the classroom. The role of peers in the learning environment was assessed using 4 items concerning interactions with peers in relation to science primarily outside of the classroom. See Appendix A for a list of the items. Item scoring was reversed where necessary so that a higher score denoted a more favourable environment. The scales were calculated as a mean of the relevant items (requiring 50% of the items to be non-missing), and demonstrated good internal consistency reliability in TEDS (alpha = 0.79 for the total; 0.71 for classroom; and 0.83 for peers). These alphas are comparable with those from the original Fraser and Kahle (2007) analysis (0.73 for classroom; and 0.79 for peers).

#### 2.2.2 Science enquiry test scores

Scientific enquiry skills, the skills needed to design and evaluate scientific evidence, are a key component in the UK National Curriculum. Our online test of scientific enquiry skills consisted of 39 items drawn from publicly available measures of science performance before being piloted and converted to web-based format. The test takes 23 min to complete on average, and has good internal consistency reliability (alpha = 0.75). A total score was calculated by taking a sum of the items. Further details about the design and piloting of our science test can be found in Haworth, Dale, and Plomin (2010). We have previously assessed the reliability and validity of our web-based tests in general by comparing web-based scores with more traditional paper-and-pencil tests. Results indicate correlations on average of 0.76 between web and paper versions (Haworth et al., 2007).

### 2.3 Analyses

#### 2.3.1 Data preparation

All of the measures were slightly skewed: the classroom environment measure had a skew of − 0.471, the peer environment 0.581, and the science test score − 0.868. A van der Waerden rank transformation (Lehmann, 1975) was applied to all of the measures. In addition, as is standard in twin analyses, all measures were corrected for the mean effects of age and sex using a regression procedure (McGue & Bouchard, 1984).

#### 2.3.2 Twin analyses

Twin analyses allow the estimation of the relative contributions of genes and environments to individual differences in measured traits (Plomin, DeFries, Knopik, & Neiderhiser, 2012). Twin intraclass correlations were calculated (Shrout & Fleiss, 1979), providing an initial indication of the relative contributions of additive genetic (A), shared environmental (C), and non-shared environmental (E) factors. Additive genetic influence, also commonly known as heritability, is estimated as twice the difference between the identical and fraternal twin correlations. The contribution of the shared environment, which makes members of a family similar, is estimated as the difference between the identical twin correlation and heritability. Non-shared environments, (environments specific to individuals), are estimated by the difference between the identical twin correlation and 1 because they are the only source of variance making identical twins different. Estimates of the non-shared environment also include measurement error.

Structural equation model-fitting analyses allow more complex analyses and formal tests of significance (Rijsdijk & Sham, 2002). Standard twin model-fitting analyses were conducted using Mx (Neale, Boker, Xie, & Maes, 2006). Sex differences in the genetic and environmental influences were examined using standard sex-limitation analyses (Neale & Maes, 2001). These models allow the formal investigation of both quantitative sex differences (i.e. different levels of effect in males and females) and qualitative sex differences (i.e. different kinds of effects in males and females).

To investigate the links between the learning environment and science performance, we conducted bivariate twin model-fitting. Bivariate model-fitting decomposes the covariance between traits, providing estimates of the genetic and environmental correlations between traits. In addition the proportion of the phenotypic correlation between the two traits that is explained by genetic and environmental factors can be computed.

## 3 Results

### 3.1 Descriptive statistics

The means and standard deviations for all of the measures are presented in Table 1. ANOVA was used to assess the effects of sex and zygosity, prior to the age and sex regression. The main effect of sex on science performance just reached significance, with boys performing better than girls, but explained only 0.2% of the variance. There was no significant effect of sex on the overall science-learning environment, but there was a significant sex difference on the peer environment scale. Males had a more favourable peer environment than females, but this effect explained only 0.4% of the variance. There were no significant main effects of zygosity. All measures were corrected for sex before the model-fitting analyses to control for these mean differences.

The phenotypic correlation between the environmental subscales was moderate (0.37), and the correlations between the environmental measures and the science test scores were modest, 0.18 for the classroom, 0.19 for the peers, and 0.23 for the overall learning environment composite (p < 0.01 for all correlations).

### 3.2 Twin correlations

The twin intraclass correlations are shown in Table 2. In all cases identical (monozygotic, MZ) twin correlations were greater than fraternal (dizygotic, DZ) twin correlations, indicating genetic influence on the measures, including the measures of the learning environment. Twin correlations split by sex, indicate that genetic and environmental estimates are similar for males and females; this question is tested formally in model-fitting analyses below.

### 3.3 Model-fitting analyses

Results from the sex-limitation analyses are shown in Table 3. For all measures the model-fitting analyses confirmed that there were no significant quantitative or qualitative sex differences. Estimates for the genetic and environmental influences from the best-fitting null model (i.e. not modelling sex differences) are shown in Table 4, along with their corresponding 95% confidence intervals. Moderate heritability was found for both scientific enquiry test scores (50%) and the science-learning environment (43%). Of note, there were negligible shared environmental influences on the science-learning environment, with the majority of the remaining variance being explained by non-shared environmental influences (54%).

Separate analyses for the classroom environment and the peer environment are also shown in Tables 3 and 4. The results for these subscales are highly similar, and multivariate analyses indicated almost complete genetic overlap between the peer and classroom scales (genetic correlation 0.98, 95% CI: 0.84–1.00; full details available from the first author). For this reason, the multivariate analyses on the relationship between science performance and the environment are presented only for the overall science-learning environment composite, but analyses using the separate environmental measures are available from the first author upon request.

**Fig. 1:**
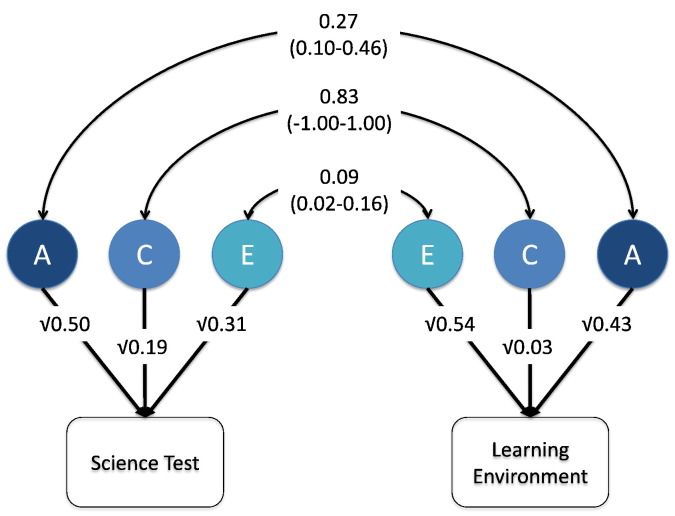
Bivariate model between test and environment. Note. A = additive genetic; C = shared environment; E = non-shared environment; 95% confidence intervals in parentheses for the genetic and environmental correlations between the test and environment. 95% confidence intervals for the ACE estimates can be found in Table 4. The 95% confidence interval for the shared environment correlation is very large because of the small variance attributed to shared environmental influence, especially on the learning environment measure. We had the option of dropping the non-significant shared environmental component for the learning environment measure. We decided not to do this because the full ACE model provides the most accurate point estimates for all of the parameters. Dropping the shared environment for the learning environment measure would have meant also dropping the shared environmental overlap between the learning environment and science performance, and we felt that it was important to include all of the environmental parameters in the analysis of the overlap between an environmental variable (the science learning environment) and the outcome (science performance). Finally, the genetic and environmental correlations must be interpreted with caution because of the low phenotypic correlation of 0.225 between these two measures.

Results from the bivariate analyses are shown in Fig. 1, which also includes the 95% confidence intervals. We found a moderate genetic correlation (0.27), indicating that to some extent the genetic influences on the learning environment also influence test performance. Of note, there was almost no overlap in non-shared environmental influences (non-shared environmental correlation = 0.09). There was strong overlap in the shared environmental influences (0.83), however, this should be interpreted in light of the minimal impact of the shared environment on the science-learning environment measure.

It is possible to calculate the contribution of genetic and environmental influences to the phenotypic correlation from the estimates in Fig. 1. The phenotypic correlation is calculated as the sum of the paths linking the two phenotypes: (√0.50 × 0.27 × √0.43) + (√0.19 × 0.83 × √0.03) + (√0.31 × 0.09 × √0.54) = 0.225. The genetic contribution to the phenotypic correlation can then be calculated: (√0.50 × 0.27 × √0.43) / 0.225 = 0.56. Thus 56% of the phenotypic correlation of 0.225 is explained by genetic influences. Similar calculations for the shared and non-shared environmental links indicate that shared environments explain 28% of the 0.225 correlation and non-shared environments explain the remaining 16%.

## 4 Discussion

The finding that the science-learning environment is modestly associated with science achievement replicates previous results (Fraser & Kahle, 2007). However, in contrast to previous findings, we find that the peer environment is just as important as the classroom environment, with both class and peer measures showing a similar level of overlap with science performance. Our main focus, however, is to extend previous research by conducting genetically sensitive analyses of the overlap between environment and outcome.

### 4.1 Twin analyses

Univariate analyses indicated that the science-learning environment was significantly heritable (43%), with minimal shared environmental influence (3%) and moderate non-shared environmental influence (54%). Results were strikingly similar for the separate subscales of the classroom environment and the peer environment. For test performance, genetic influences explained 50% of the variance and shared and non-shared environments explained 19% and 31%, respectively. These results for test data confirm previous analyses of science performance as rated by teachers (Haworth et al., 2009). No significant sex differences were detected for genetic and environmental influences on the environmental measures or the test, indicating that the same genes and environments impact males and females and that genetic and environmental effect sizes are the same for males and females.

Bivariate twin analyses indicated that 56% of the phenotypic correlation between the science-learning environment and science performance was explained by genetic influences. Environmental influences explain the remaining overlap, with shared environments explaining more of the relationship (28%), than non-shared environments (16%). However, note that because the phenotypic correlation is only 0.225, this means that overlapping genetic factors explain just a small proportion (2.8%) of the *total* variance in science performance. The main reason why there is a correlation between environment and outcome is shared genetic influences. This gene–environment correlation is characteristic of other analyses between environments and outcomes (Walker & Plomin, 2006), and indicates that genetically influenced behaviours have an impact on our experience of the environment.

### 4.2 Limitations

Although the phenotypic correlation (0.23) is in line with previous studies that found an average correlation of 0.13 for individual analyses, it is still only modest, indicating that the science-learning environment explains only a small proportion of the variance in science performance. This low correlation warrants some caution in interpreting the multivariate analyses. There are at least two (not mutually exclusive) explanations for the low correlation: that the quality of the science-learning environment is not a good predictor of science performance, or that an individual's perception of their learning environment is not a good indicator of the quality of the environment. We cannot unequivocally rule out either of these possibilities, however the learning environment questionnaire is well validated (Fraser & Kahle, 2007), and the questionnaire demonstrates good internal consistency reliability in TEDS. The learning environment measure also shows relatively high heritability for an ‘environmental’ measure — and heritability is capped by the reliability of the measure.

The modest overlap between the learning environment, and in particular the classroom environment, and science performance is consistent with other studies that have attempted to quantify the effect that teachers have on student outcomes (e.g., Byrne et al., 2010). These empirical findings are often at odds with the popular press stories about bad teachers and the detrimental effects of poor teaching quality. Although our results do not speak directly to the issue of teacher effects on performance, they do highlight the fact that interactions between students and teachers (and students and peers) may create highly individualised experiences of these learning environments. This means that there are likely to be as many different classroom environments as there are students in the class. Educational researchers must now acknowledge that classroom (and peer) effects should be studied at the individual level as well as at the aggregate classroom level to fully understand the dynamic learning environments created by person–situation and person–person interactions.

More generally, our findings highlight the difficulties in identifying environments that matter, especially at the level of the individual. Future studies should consider multiple measures of the environment that individually explain a small proportion of the variance, but when combined into an environmental index, can explain larger proportions of the variance.

A further potential limitation is the use of perceptions of the environment rather than observer ratings. We are not aware of any genetically sensitive investigations of observer ratings in the classroom, but genetic influences have been found for observer ratings of the home environment (O'Connor, Hetherington, Reiss, & Plomin, 1995), suggesting that the genetic influence is not simply a by-product of using self-ratings of experience. Finally, although members of each twin pair were in the same school, we do not have information about whether the twins were in the same or different science classrooms at age 14. However, we do know that similar proportions of MZ and DZ twins are in the same classroom at earlier ages in TEDS (Kovas et al., 2007), suggesting that differences in classroom sharing are unlikely to explain the differences in the twin correlations for MZ and DZ twins.

### 4.3 Conclusions

The science-learning environment shows genetic influence, indicative of gene–environment correlation, whereby individuals create, seek out or perceive environments that are correlated with their genetic propensities (Haworth, Asbury, Dale, & Plomin, 2011; Haworth, Wright, et al., 2010). Educational policy needs to acknowledge that the school environment is not something that just passively happens — rather humans create their own environments to a large extent, and evoke reactions from their environments (Plomin & Bergeman, 1991; Scarr & McCartney, 1983). Children bring both their genetic and environmental backgrounds to the classroom. They elicit responses from their teachers and peers, and select particular peers in part because of genetic propensities, shaping their educational experience, and impacting their school performance.

## Figures and Tables

**Table:** 

Answer options: almost never; seldom; sometimes; often; very often
1. My teacher asks questions that have more than one answer.
2. My teacher asks me to give reasons for my answers.
3. My teacher encourages me to ask questions.
4. I learn from my classmates.
5. I use information to support my answers.
6. I talk to my classmates about how to solve problems.

**Table:** 

Answer options: almost never; seldom; sometimes; often; very often
1. My friends talk about Science outside of class.
2. My friends discuss things they have learned in Science.
3. My friends enjoy doing Science-related activities outside of class.
4. My friends are interested in Science.

**Table 1:** Means (standard deviations) and ANOVA results.

	Science test	Learning environment	Classroom environment	Peer environment
All	0.01	0.01	0.01	0.00
	(1.00)	(0.99)	(0.99)	(1.00)
	N = 2741	N = 3196	N = 3195	N = 3188
MZ	− 0.03	0.01	0.01	0.02
	(1.00)	(1.02)	(1.00)	(1.03)
	N = 1059	N = 1196	N = 1196	N = 1192
DZ	0.04	0.00	0.02	− 0.01
	(1.00)	(0.97)	(0.99)	(0.98)
	N = 1682	N = 2000	N = 1999	N = 1996
Male	0.06	0.03	− 0.01	0.07
	(1.00)	(1.02)	(1.02)	(1.00)
	N = 1144	N = 1426	N = 1426	N = 1421
Female	− 0.03	− 0.01	0.04	− 0.06
	(1.00)	(0.97)	(0.96)	(0.99)
	N = 1597	N = 1770	N = 1769	N = 1767
Sex p-value	0.016	0.205	0.229	< 0.001
Sex effect size	0.002	0.001	< 0.001	0.004
Zygosity p-value	0.162	0.633	0.818	0.273
Zygosity effect size	0.001	< 0.001	< 0.001	< 0.001

Note. N = one randomly selected member of each pair; all measures transformed; effect size expressed as eta squared. MZ = monozygotic twins; DZ = dizygotic twins. Standardised scores are presented because the measures were rank transformed to adjust for skew prior to the ANOVA.

**Table 2:** Intraclass twin correlations by sex and zygosity.

	Science test	Learning environment	Classroom environment	Peer environment
MZ	0.67	0.47	0.40	0.45
	N = 943	N = 1149	N = 1149	N = 1146
DZss	0.45	0.21	0.19	0.25
	N = 729	N = 992	N = 991	N = 989
DZos	0.42	0.26	0.22	0.23
	N = 653	N = 873	N = 873	N = 868
DZall	0.44	0.24	0.20	0.24
	N = 1382	N = 1865	N = 1864	N = 1857
MZM	0.65	0.52	0.46	0.45
	N = 366	N = 490	N = 490	N = 487
MZF	0.68	0.43	0.35	0.45
	N = 577	N = 659	N = 659	N = 659
DZM	0.43	0.14	0.13	0.19
	N = 310	N = 446	N = 446	N = 444
DZF	0.47	0.27	0.24	0.29
	N = 419	N = 546	N = 545	N = 545

Note. N = number of complete twin pairs. MZ = monozygotic twins; DZss = dizygotic same-sex twins; DZos = dizygotic opposite-sex twins; DZall = all dizygotic twins (same-sex and opposite-sex combined); MZM = monozygotic male twins; MZF = monozygotic female twins; DZM = dizygotic male twins; DZF = dizygotic female twins. Two of the items in the classroom scale refer to ‘classmates’, and because students may have more influence on their peers, and therefore increase the influence of their genes on the environmental measure, we repeated the analyses dropping the two classmate items. Results were very similar for the classroom scale with and without these items, with rMZ = 0.36 and rDZ = 0.21 for the reduced classroom scale.

**Table 3:** Sex limitation fit statistics for test and environment.

Measure	Model	− 2LL	*df*	AIC	LRT	Δ*df*	p
Test	1. Full (r_G_/r_C_ free)	14,640.183	5452	3736.183	25.395	12	0.013^a^
	2. Common effects	14,640.410	5453	3734.410	0.226	1	0.634
	3. Scalar	14,641.197	5455	3731.197	1.013	3	0.798
	4. Null model	14,647.013	5456	3735.013	6.829	4	0.145
Learning environment	1. Full (r_G_/r_C_ free)	17,723.810	6377	4969.810	19.685	12	0.073^a^
	2. Common effects	17,723.810	6378	4967.810	0.000	1	1.00
	3. Scalar	17,728.228	6380	4968.228	4.418	3	0.220
	4. Null model	17,730.689	6381	4968.689	6.879	4	0.142
Classroom environment	1. Full (r_G_/r_C_ free)	17,828.416	6375	5078.416	14.086	12	0.295^a^
	2. Common effects	17,828.416	6376	5076.416	0.000	1	1.00
	3. Scalar	17,833.424	6378	5077.424	5.008	3	0.171
	4. Null model	17,836.205	6379	5078.205	7.789	4	0.100
Peer environment	1. Full (r_G_/r_C_ free)	17,706.947	6362	4982.947	13.118	12	0.361^a^
	2. Common effects	17,706.947	6363	4980.947	0.000	1	1.00
	3. Scalar	17,709.923	6365	4979.923	2.976	3	0.395
	4. Null model	17,710.762	6366	4978.762	3.814	4	0.432

Note. Full model = this model allows quantitative and qualitative sex differences as well as different variances for males and females; common effects model = this model allows quantitative sex differences and different variances for males and females; scalar model = this model only allows different variances for males and females; null model = this model allows no sex differences. Common effects, scalar and null models are compared to the fit of the full model. The full model is compared to the fit of the saturated model (^a^ = compared to the saturated model with MZ = DZ mean and twin 1 = twin 2 means). − 2LL = minus twice the log likelihood; *df* = degrees of freedom; AIC = Akaike's information criterion (lower values indicate better fit); LRT = likelihood ratio test (change in likelihood between two models distributed as chi-squared); Δ*df* = change in degrees of freedom between comparison models; p = p-value for LRT.

**Table 4:** Univariate estimates (and 95% confidence intervals) for genetic, shared environment and non-shared environment.

	Genetic	Shared environment	Non-shared environment
Science test	0.50	0.19	0.31
	(0.40–0.59)	(0.10–0.27)	(0.29–0.35)
Learning environment	0.43	0.03	0.54
	(0.31–0.50)	(0.00–0.12)	(0.50–0.59)
Classroom environment	0.37	0.02	0.61
	(0.25–0.44)	(0.00–0.12)	(0.56–0.65)
Peer environment	0.39	0.05	0.56
	(0.27–0.48)	(0.00–0.15)	(0.52–0.61)

Note. Genetic and environmental influences are from the best-fitting univariate analyses (the null model in Table 3).

## Appendix A Items included in the science-learning environment scales

### A) Science classroom

In my Science classes…

### B) Peer environment

In relation to Science…

Note: the original questionnaire (Fraser & Kahle, 2007) included 7 and 5 items respectively for the class and peer environments. We dropped the item ‘I repeat experiments to check results’ from the class scale, and the item ‘my friends work on science projects’ from the peer scale. This was primarily to ensure consistency in the items we included for assessing the learning environment in other subjects (e.g. English and Mathematics).
